# Investigating the Spatial Distribution and Influencing Factors of Non-Grain Production of Farmland in South China Based on MaxEnt Modeling and Multisource Earth Observation Data

**DOI:** 10.3390/foods13213385

**Published:** 2024-10-24

**Authors:** Juntao Chen, Zhuochun Lin, Jinyao Lin, Dafang Wu

**Affiliations:** 1 School of Geography and Remote Sensing, Guangzhou University, Guangzhou 510006, China; 2112201082@e.gzhu.edu.cn (J.C.); 32201500009@e.gzhu.edu.cn (Z.L.); wudafang@gzhu.edu.cn (D.W.); 2 Huangpu Research School of Guangzhou University, Guangzhou 510006, China

**Keywords:** food security, non-grain production, agricultural development, farmland protection, influencing factor

## Abstract

Excessive non-grain production of farmland (NGPF) seriously affects food security and hinders progress toward Sustainable Development Goal 2 (Zero Hunger). Understanding the spatial distribution and influencing factors of NGPF is essential for food and agricultural management. However, previous studies on NGPF identification have mainly relied on high-cost methods (e.g., visual interpretation). Furthermore, common machine learning techniques have difficulty in accurately identifying NGPF based solely on spectral information, as NGPF is not merely a natural phenomenon. Accurately identifying the distribution of NGPF at a grid scale and elucidating its influencing factors have emerged as critical scientific challenges in current literature. Therefore, the aims of this study are to develop a grid-scale method that integrates multisource remote sensing data and spatial factors to enhance the precision of NGPF identification and provide a more comprehensive understanding of its influencing factors. To overcome these challenges, we combined multisource remote sensing images, natural/anthropogenic spatial factors, and the maximum entropy model to reveal the spatial distribution of NGPF and its influencing factors at the grid scale. This combination can reveal more detailed spatial information on NGPF and quantify the integrated influences of multiple spatial factors from a microscale perspective. In this case study of Foshan, China, the area under the receiver operating characteristic curve is 0.786, with results differing by only 1.74% from the statistical yearbook results, demonstrating the reliability of the method. Additionally, the total error of our NGPF identification result is lower than that of using only natural/anthropogenic information. Our method enhances the spatial resolution of NGPF identification and effectively detects small and fragmented farmlands. We identified elevation, farming radius, and population density as dominant factors affecting the spatial distribution of NGPF. These results offer targeted strategies to mitigate excessive NGPF. The advantage of our method lies in its independence from negative samples. This feature enhances its applicability to other cases, particularly in regions lacking high-resolution grain crop-related data.

## 1. Introduction

Food security is a worldwide issue that considerably threatens human livelihoods and undermines the achievement of the Sustainable Development Goal (SDG) 2: Zero Hunger [1,2,3]. Farmland serves as an irreplaceable foundation for food production and is therefore closely related to food security [4,5,6]. In many countries, however, a large amount of farmland has not been used for growing major grain crops. This situation is particularly serious in China. Despite accounting for approximately 17.6% of the global population, China has only 7.8% of the world’s farmland [7,8]. Between 1995 and 2015, built-up areas in eastern China expanded by 12,700 km^2^, with over two-thirds of this growth (8600 km^2^) resulting from the conversion of farmland [9]. Furthermore, between 2010 and 2020, China experienced a decline of 75,330 km^2^ in farmland area, posing a significant challenge to national food security [10].

In fact, rice, wheat, and corn are the three major grain crops in China, collectively accounting for 92.45% of the total grain production in 2019, according to data from the China Statistical Yearbook. Therefore, safeguarding the cultivation of these three major grain crops is essential for ensuring food security. Nevertheless, a significant portion of farmland in China is not utilized for the cultivation of these major grain crops (rice, wheat, and corn). According to previous studies, alternative cultivation or utilization practices on farmland other than the planting of the three major grain crops are defined as “non-grain production of farmland” (NGPF) [11,12,13]. These practices include the cultivation of cash crops (vegetables and fruits), engagement in forestry, and the construction of ponds for aquaculture [14,15,16].

The increasing proportion of NGPF indicates a decreasing allocation of farmland to grain production, which poses a significant threat to grain food security. Notably, the prevalence of NGPF has been gradually increasing nationwide with ongoing socioeconomic development [17,18,19]. Therefore, NGPF has received extensive attention from policymakers and scholars in related fields [20,21,22,23]. In general, most previous studies analyzed the spatial distribution of NGPF only at large scales [24,25,26,27]. Specifically, administrative regions (e.g., cities or counties) were usually adopted as the basic units in those attempts [7,28,29]. This is because they relied heavily on the data collected from statistical yearbooks, such as the total farmland area and grain crop planting area [30,31,32,33]. Unfortunately, these types of data are insufficient for fine-scale spatial analysis of NGPF because they cannot capture the spatial heterogeneity necessary for the formulation of effective food security policies at the local level.

In fact, accurate identification of NGPF is fundamental for understanding grain planting conditions and preventing excessive NGPF [34,35,36]. A fine-scale spatial analysis can provide much more detailed information on food security [37,38]. To this end, several earlier studies have identified NGPF at grid scales based on visual interpretations of high-resolution remote sensing images [12,39]. For instance, Chang et al. [40] conducted a visual interpretation of satellite imagery with a resolution of 0.8 m to delineate NGPF patches, which were subsequently validated through UAV-based field verification. Nevertheless, this method is high-cost and inefficient [37,41,42]. Similar attempts have been made based on farmland information from China’s National Land Survey, but such surveys were still based on extensive field investigations. Although machine learning techniques have been increasingly used in recent years to identify farmland based on high-resolution remote sensing images [43,44], NGPF cannot be accurately identified simply based on spectral information given its complexity. In addition, considerable uncertainties are involved during sample collection [39,45].

Interestingly, the maximum entropy (MaxEnt) model can effectively address the limitations of previous studies. The MaxEnt model was originally proposed by Jaynes [46] based on the principle of information entropy and can yield high accuracy, especially when the training samples are limited. Compared with commonly used ground object recognition methods, such as decision trees, support vector machines, random forests, and neural networks, the key advantage of MaxEnt is that it can provide desirable modeling results when only positive samples are used. MaxEnt was initially used to predict the spatial distribution of species [47,48]. MaxEnt has also been widely used in environmental research to identify grasslands [49] and conservation areas [50]. However, it is worth noting that the MaxEnt model has rarely been employed in studies focused on NGPF identification, which has the potential to significantly improve the precision of identification.

Another severe concern is that previous studies have paid more attention to qualitative influencing factors of NGPF, such as the characteristics of farmers, social capital investment, and agricultural policies [33,51,52,53]. For example, Li et al. [30] reported that low income from grain crop planting is the main reason for the occurrence of NGPF. Niu et al. [54] noted that the age and gender of farmers have an impact on the occurrence of NGPF. However, in addition to the aforementioned macroscale qualitative factors, microscale spatial factors such as natural and anthropogenic conditions play an even more important role [55,56]. In summary, the research gap in the literature can be attributed to two main aspects. Firstly, there is a lack of fine-scale spatial identification of NGPF, especially at the grid scale. Secondly, there has been limited application of advanced models such as MaxEnt, which can accurately identify NGPF using only positive samples and spatial factors. The majority of previous studies have concentrated on large-scale analysis, thereby overlooking the microscale spatial heterogeneity of NGPF and the interaction of spatial factors at the grid scale.

To fill the above gaps in the literature, this study will investigate the following research questions: What is the spatial distribution of NGPF in the study area at a grid scale? What specific natural and anthropogenic factors drive NGPF from the perspective of grid scale? We hypothesize that a grid-scale analysis combined with multisource spatial data will significantly enhance the precision of NGPF identification and provide new insights into the mechanisms by which these spatial factors influence NGPF. This approach differs significantly from previous research that has primarily focused on large-scale analysis. To this end, our study employed the MaxEnt model to investigate the spatial pattern and influencing factors of NGPF at the grid scale based on Landsat 8 and Sentinel-2 images, land cover data, and grain crop planting data. In fact, the combination of high-resolution remote sensing images, natural/anthropogenic spatial factors, and the MaxEnt model offers two major advantages. First, the incorporation of natural/anthropogenic factors can reveal more detailed spatial information on NGPF because it is not just a natural phenomenon. Second, the integrated influences of multiple factors can be effectively analyzed from a microscale perspective when only positive samples are used. This approach can overcome the limitations of previous studies, which relied heavily on the quality of both positive and negative samples for accuracy. The results are expected to provide practical decision support for the prevention and management of NGPF. Therefore, the aims of this study are to accurately identify the spatial distribution of NGPF at the grid scale and to elucidate the joint impacts of both natural and anthropogenic factors, thereby providing practical decision support for the prevention and management of NGPF.

## 2. Materials and Methods

### 2.1. Study Area

The city of Foshan, where the NGPF problem is prominent, was selected as the study area. Foshan is located in the middle of Guangdong Province, China. The overall NGPF rate of Guangdong Province exceeded 45% in 2018 [37], and rice production in this region declined by 5.61 million tons from 1998 to 2016, the second largest decline in China [57]. The NGPF issue is particularly severe in Foshan [58]. This city covers a total area of 3797 km^2^ and has a humid subtropical monsoon climate with sufficient precipitation. The gross domestic product (GDP) of Foshan increased from 238.32 billion CNY to 1075.10 billion CNY from 2005 to 2019, whereas its farmland area decreased from 559.05 km^2^ to 211.64 km^2^. Moreover, the proportion of NGPF in Foshan reached 86.18% in 2019 according to the Foshan Statistical Yearbook. Approximately 78.57% of the NGPF in Foshan was used for growing vegetables. Although it is well accepted that socioeconomic conditions have an enormous impact on NGPF issues, many other factors are impossible to consider in macroscale research, for example, taking an administrative region as the research unit [59]. Therefore, it is important to investigate the fine-scale spatial influencing factors of NGPF in this city.

### 2.2. Data Sources and Processing

#### 2.2.1. Data Sources

First, the remote sensing images used in this study included Landsat 8 OLI/TIRS and Sentinel-2 images from 2019. The Landsat images were obtained from the Geospatial Data Cloud Platform of China. Given the key phenological information of crop planting in the study area, two scenes of cloud-free and high-quality images in September and October were selected. In addition, the Sentinel-2 data came from the European Space Agency, and images with no or limited cloud coverage for every month were selected.

Second, the crop-related data included the planting areas for three major grain crops (with a 1 km spatial resolution) in 2019 from the National Ecosystem Science Data Center of China [60], and the rice planting areas in 2019 (with a 500 m spatial resolution) [45].

Third, the potential influencing factors of NGPF included roads (vectors) provided by OpenStreetMap; the spatial distribution of rural settlements in 2018 at 30 m resolution from the Chinese Academy of Sciences; the China Land Cover Dataset (CLCD) in 2019 at 30 m resolution from Wuhan University [61]; temperature, precipitation, population, and GDP in 2019 from the National Earth System Science Data Center; the digital elevation model (DEM) from the Geospatial Data Cloud Platform; and the comprehensive index of land use degree (CILUD) and residential land density from the Scientific Data Bank [62].

Finally, two types of potential spatial influencing factors (natural and anthropogenic conditions) were generated based on previous findings [63,64,65,66,67,68] and the local characteristics of Foshan. Specifically, previous studies have indicated that common natural factors include elevation, slope, slope aspect, precipitation, and temperature [64,65], whereas typical anthropogenic factors include GDP and population density [63]. Additionally, actual evapotranspiration, potential evapotranspiration, and rainfall erosivity are crucial factors for crop growth [68]. Moreover, factors such as the farming radius, traffic conditions, and irrigation conditions could significantly impact NGPF [65]. An excessive farming radius or poor traffic and irrigation conditions may reduce farmers’ interest in growing grain crops, which could lead to NGPF [68]. Furthermore, irrational CILUD and residential land density could induce the conversion of farmland to cash crop cultivation or changes in land use practices [69]. Table S1 (in the Supplementary Materials) presents detailed information (e.g., spatial resolution, years) on these factors. Since crop planting may most likely be affected by long-term conditions, multiyear average data for several key factors were adopted. Specifically, 30-year (1991–2020) averages were applied, including annual average precipitation, annual average temperature, potential evapotranspiration, actual evapotranspiration, and rainfall erosivity. These data were provided by the Chinese Academy of Sciences at a 30 m resolution.

#### 2.2.2. Data Processing

**Figure 1:**
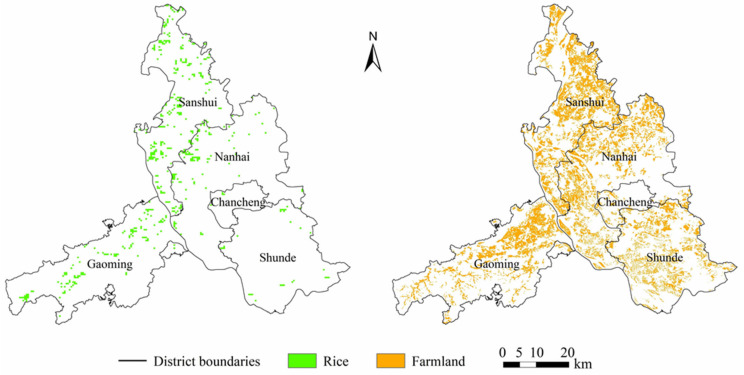
Spatial distributions of rice planting and farmland in Foshan (the sources for rice planting and farmland are Luo et al., 2020 [60] and Han, J. et al., 2022, [45] respectively).

First, both the Landsat 8 and Sentinel-2 images were processed through georeferencing, atmospheric correction, topographic correction, etc. Next, the spatial distribution of farmland was extracted from the CLCD. According to local investigations and statistical data, no wheat or corn is grown in Foshan. Therefore, only rice was considered a grain crop in this case study. The spatial distributions of rice planting and farmland in Foshan are shown in Figure 1. These two datasets were overlaid to reveal a rough spatial coverage of NGPF, in which a specified number of samples (200, 500, 750, 1000, 2000, 5000, and 10,000) were randomly selected for building the MaxEnt models. Eventually, we found that the highest AUC score could be obtained by MaxEnt when the sample number was 500, and this best result was adopted for further analysis.

**Figure 2:**
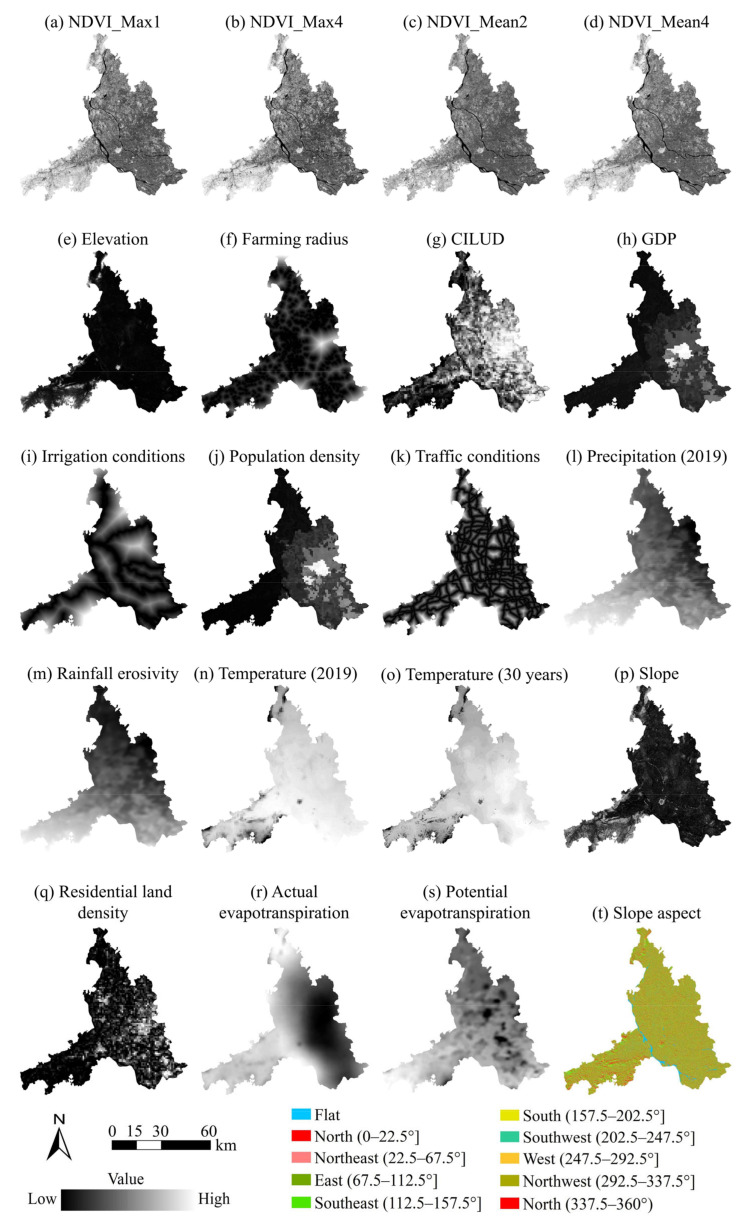
The remaining spatial influencing factors of NGPF.

Second, the pixel values for each band of the Landsat images were extracted, and the Sentinel-2 images were used to generate the maximum and mean normalized difference vegetation indices (NDVIs) for each season in 2019. To prevent the negative influence of multi-collinearity, a Pearson correlation analysis was performed on all the influencing factors mentioned above. The factors with a lower contribution rate in MaxEnt were excluded if the correlation coefficient between them was greater than 0.7 [70]. Twenty-five factors (Figure 2) remained after this operation.

### 2.3. Maximum Entropy (MaxEnt)

MaxEnt has been widely used in numerous fields, such as species distribution modeling and ground object recognition [49,65,71,72,73]. This model can yield desirable accuracies with limited training samples and low computational cost. The spatial distribution of ground objects (e.g., species) can be accurately identified based on given sample points of ground objects and associated constraints (e.g., influencing factors). The versatility and effectiveness of MaxEnt have made it a valuable tool in a variety of contexts, demonstrating its capacity to deal with complex interactions between variables and geographical challenges. For instance, Zhou et al. [74] employed the MaxEnt method to evaluate the suitability of rural settlements in the farming-pastoral ecotone of Northern China. They quantitatively measured the importance of 13 key influencing factors based on data from 1996, 2010, and 2020. In addition, Zuo and Zhang [75] utilized the MaxEnt model to integrate environmental variables with the locations of cultural ecosystem services (CESs) in rural areas of the Yangtze River Delta. This facilitated the mapping of spatial factors and the quantitative evaluation of the influence of each factor on local CESs. Furthermore, Wang et al. [76] employed the MaxEnt model to analyze the environmental suitability for rapeseed cultivation in Qinghai Province. This involved integrating agricultural accessibility with environmental factors to optimize crop cultivation planning in relation to ecotourism development. Assume that the set of sample points *x_i_* is *X*, *X =* {*x*_1_, *x*_2_, ..., *x_m_*}, and constraint set *F* contains a number of eigenvalues *f_i_*, *F* = {*f*_1_, *f*_2_, ..., *f_n_*}. These constraints represent incomplete information on the target distribution. Then, the probability distribution $\overset{\sim }{\pi }(x)$ of the ground object can be estimated to approximate the actual distribution π(x) as follows [48]:

(1) $$\overset{\sim }{\pi }\left(x\right)=\frac{\left|\left\{1⩽i⩽m:x_{i}=x\right\}\right|}{m}$$

where *x_i_* denotes the *i*th sample and *m* denotes the number of samples.

In addition, the mean of *f_i_* is defined as follows:

(2) $$\overset{\sim }{\pi }(f_{i})=\frac{1}{m}\sum_{i=1}^{m}f_{i}(x_{i})$$

According to the principle of MaxEnt, the distribution with the largest entropy should be selected, and the entropy π^ is defined as follows [77]:

(3) H(π^)=−∑x∈Xπ^(x)ln⁡π^(x)

Compared with other common machine learning models, the advantage of MaxEnt is that it can yield desirable accuracies without depending on negative samples (farmland where rice is planted) [49,65]. For example, the application of random forests and neural networks requires the use of both accurate positive (farmland where rice is not planted) and negative samples for accurate classification. However, due to the complexity of negative samples, their collection is susceptible to selection bias, which may result in unreliable identification outcomes when using traditional machine learning methods that heavily rely on both positive and negative samples [72,78,79]. In this study, however, the grain crop-related data were not 100% accurate. Moreover, the spatial resolution (500 m) is much coarser than that of the remote sensing images (30 m). Therefore, the accuracy of negative samples cannot be guaranteed.

In this study, the maximum entropy model was constructed using the MaxEnt 3.4.4 package. The normalized data of the 25 factors mentioned in Section 2.2.2 were input into the package, and the average of 20 repeated runs was taken as the final result. For each single run, 75% of the NGPF samples were randomly selected as training data, and the remaining 25% were used as test data. In addition, the other parameters were set to the default values (e.g., cloglog output; creating response curves; prevalence is 0.5) in light of previous findings [65,71]. Finally, the distribution probability results were separated into grain-production farmland and NGPF (i.e., the final NGPF identification result) according to the threshold rule of “Fixed Cumulative Value 10” [72,80].

In this study, two methods were adopted to examine the performance of the NGPF identification results. The first method is based on the area under the receiver operating characteristic (ROC) curve (AUC). The ROC is a curve plotted with sensitivity (the ratio of the number of correctly estimated NGPF to the number of NGPF samples selected in Section 2.2.2, i.e., the true positive rate) as the y-axis coordinate and 1-specificity (i.e., the false positive rate) as the x-axis coordinate. The AUC derived from the ROC curve is the most common method for examining the performance of MaxEnt models. The AUC ranges from 0 to 1, with a larger value indicating better performance. In general, the modeling result is considered reliable when its AUC is greater than 0.7 [74,79,81,82]. The second method is an empirical evaluation, which means that the NGPF identification result was compared with statistical yearbook data and the rough spatial coverage of the NGPF (Section 2.2.2).

**Figure 3:**
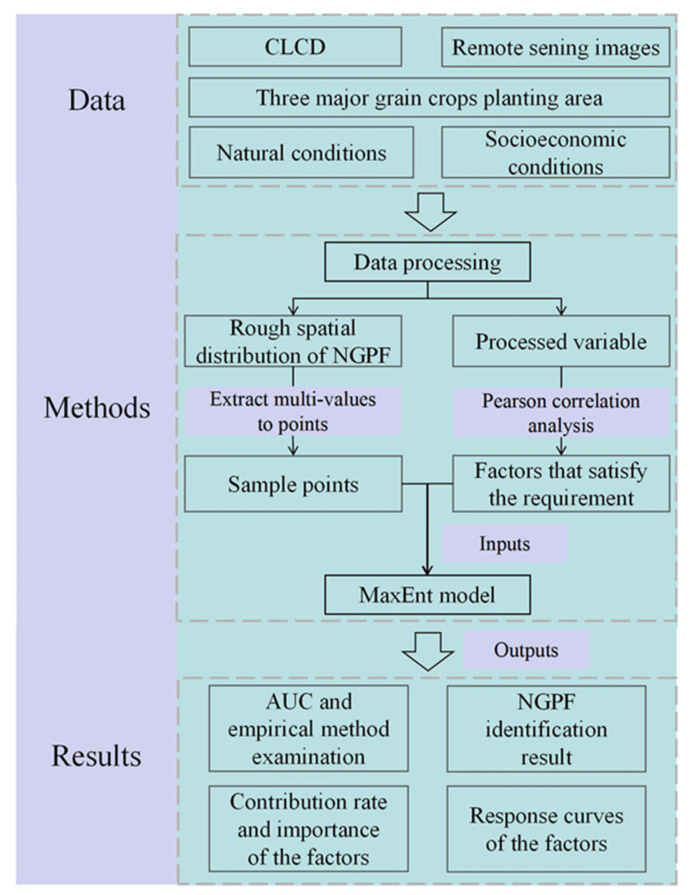
Flowchart of NGPF identification based on the MaxEnt model and multisource earth observation data.

The MaxEnt model can also measure the contribution rate, importance, and response curve of every influencing factor. The contribution rate was calculated by adding or subtracting the regularization gain from the corresponding factor during training iterations. In addition, the factor importance was evaluated by randomly permuting the values of the factor and then calculating the difference in the AUC (normalized to percentages). Moreover, the response curves reveal how the occurrence probability of NGPF changes with each factor, while the other factors remain at their mean sample values. In summary, the flowchart of this research is illustrated in Figure 3.

## 3. Implementation and Results

### 3.1. Examination of the MaxEnt Model

**Figure 4:**
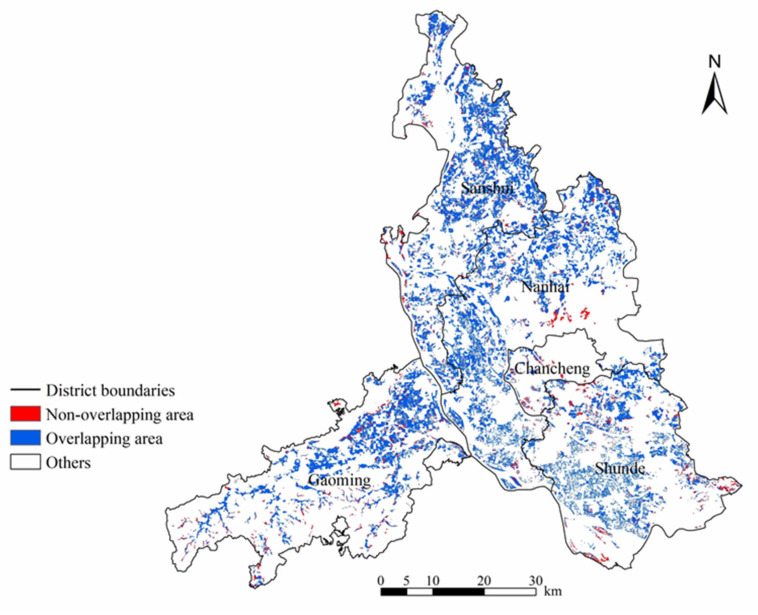
Overlapping areas between our NGPF identification results and rough NGPF data.

In this case study, the AUC of the MaxEnt model was 0.786 (Figure S1), indicating that the results are reliable. Moreover, we found that the proportion of NGPF to total farmland area calculated based on the model threshold was highly consistent with the proportion calculated based on the statistical yearbook, with a net difference of only 1.74%. In addition, we overlaid the NGPF identification results and the rough spatial coverage of the NGPF to explore the differences between them, which further demonstrated the reliability of our identification results. The overlap ratio was approximately 88.99% (Figure 4). This difference is mainly due to the inconsistent spatial resolutions of these data. That is, the spatial resolution of the rough NGPF data is merely 500 m, while our identification result has a much finer resolution (30 m). In summary, our method can considerably improve the spatial resolution of NGPF identification results, which should be much better for identifying small and fragmented cultivated land.

### 3.2. Spatial Characteristics of NGPF

**Figure 5:**
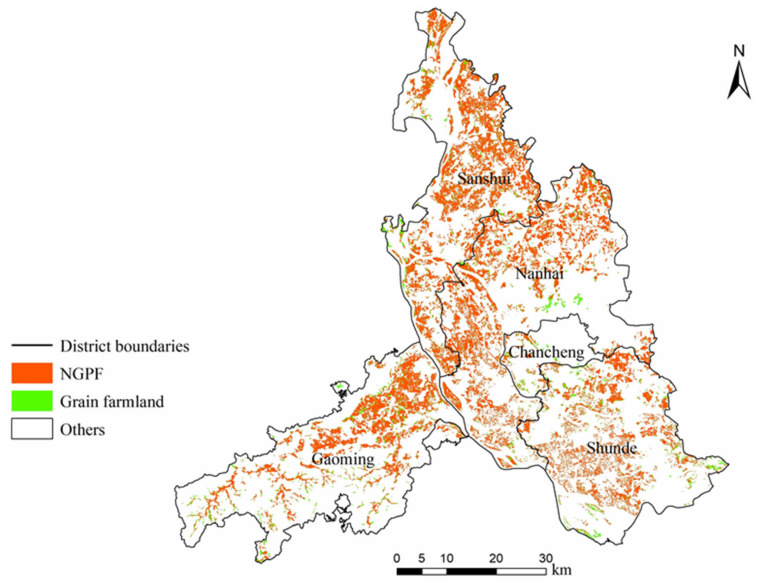
NGPF identification results based on MaxEnt.

As shown in Figure 5 and Table 1, we found that NGPF was densely distributed in the northern, central, and southeastern regions and was generally scattered across the central and western regions. The western part was not suitable for cultivation due to its high altitude and steep slope. In addition, we calculated the proportions of NGPF in each district to the total farmland of Foshan and to the total land area of the district. Sanshui District had the highest proportions, of 28.00% and 33.78%, respectively, and Chancheng District had the lowest proportions, of 0.93% and 6.05%, respectively. The reason is that Chancheng District is economically developed and has much less farmland than the other districts. Moreover, according to Table 1, the total difference between our results and the statistical yearbooks is 15.36%, of which the largest difference is in Sansui.

To further evaluate our proposed method, the data in Section 2.2.2 are divided into two types: (1) spectral information (e.g., bands, NDVI) and (2) natural/anthropogenic information. These two types of data were used to build the MaxEnt model separately, and the NGPF identification results with the highest AUC were compared with our results mentioned above. The AUCs obtained by using only the spectral information and only the natural/anthropogenic information were 0.725 (Figure S2a) and 0.736 (Figure S2b), respectively. These values are both lower than the AUC of our result (0.786). In addition, according to Table 1 and Table S1, the total difference in our NGPF identification result is smaller than the total difference in the result using only the natural/anthropogenic information. This observation also confirms that the former is more accurate.

### 3.3. Contribution Rate and Importance of Each Influencing Factor

Table 2 shows the contribution rate of each influencing factor. The five most influential factors were elevation, farming radius, CILUD, population density, and residential land density. In addition, the importance and ranking of each influencing factor are shown in Table 3, and the five factors with the greatest influence were elevation, farming radius, GDP, irrigation conditions, and actual evapotranspiration.

After considering both the contribution rate and importance, the top-ranked influencing factors in each category were regarded as the dominant factors, namely, elevation, farming radius, CILUD, and population density.

**Figure 6:**
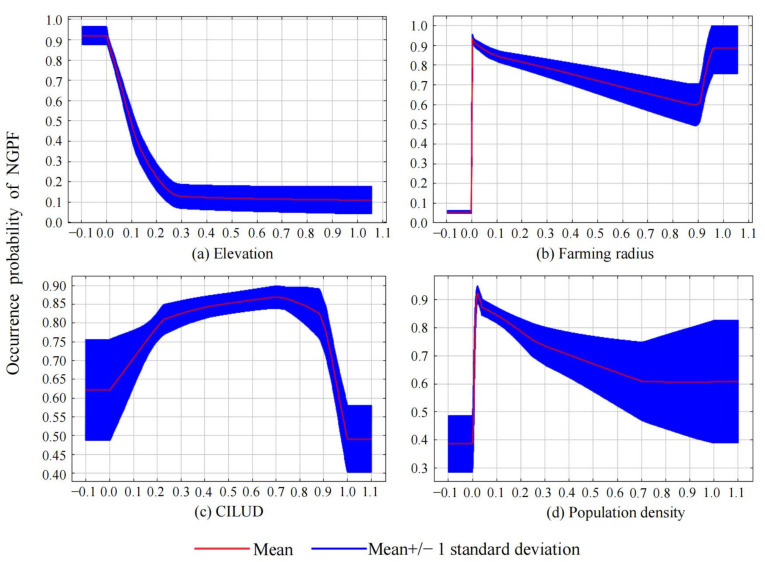
Response curves between dominant factors and occurrence probability of NGPF.

Figure 6 shows that (1) the occurrence probability of NGPF decreases with increasing elevation and finally stabilizes at approximately 0.1; (2) the probability of NGPF first decreases, then increases and finally stabilizes with increasing farming radius; (3) when the CILUD increases, the probability of NGPF increases continuously, and then begins to decline after reaching a peak when the CILUD is approximately 0.7; and (4) the probability of NGPF reaches a peak when the population density is approximately 0.02; then, the probability decreases with increasing population density and reaches the lowest point when the population density is approximately 0.7.

In addition to the above four dominant factors, residential land density, GDP, irrigation conditions, actual evapotranspiration, and slope were also important factors affecting the spatial distribution of NGPF (Figure S3).

## 4. Discussion

### 4.1. Comparison Between the Proposed Method and Traditional Methods

In general, the methodologies adopted by previous NGPF-related studies can be summarized into two categories. The first category builds upon socioeconomic statistical yearbooks, and thus, the spatial pattern and influencing factors of NGPF were analyzed only with administrative regions as basic units. In particular, “county” has been widely adopted as a basic unit for analyzing the spatial characteristics of NGPF [83,84]. Unfortunately, such methods cannot reveal detailed information on NGPF at a finer scale.

Second, remote sensing images are increasingly used in fine-scale NGPF research. For example, Su et al. [12] identified NGPF through visual interpretations of high-resolution remote sensing images. Since this method is time-consuming, a number of studies have also combined machine learning with high-resolution remote sensing images to identify ground objects. However, NGPF cannot be accurately identified based only on spectral information because it is not just a natural phenomenon. Moreover, the performance cannot be guaranteed due to a heavy dependence on the accuracies of both positive and negative samples [65,81]. Considerable uncertainties are involved during sample collection.

The MaxEnt model adopted in this study can provide desirable ground object identification results when only positive samples are used. This advantage can minimize the burden of collecting negative samples required by other conventional machine learning techniques. More importantly, the combined use of multisource remote sensing images and natural/anthropogenic spatial factors can overcome the limitation of using only spectral information. Although the proposed method was tested based only on data from 2019 in Foshan, it is also applicable to other regions and periods, especially when high-resolution and accurate grain crop-related data (e.g., planting areas for major grain crops) are unavailable.

Our method offers several tangible benefits to companies and governmental land management departments. First, our method eliminates the need to collect negative samples, allowing practitioners to focus on collecting positive samples. This streamlines the identification process of NGPF, resulting in significant time and manpower savings for organizations or companies engaged in farmland management. Second, by combining multisource remote sensing images with natural and anthropogenic spatial factors, our method enhances the accuracy and robustness of MaxEnt compared to methods that rely solely on spectral information. By integrating data inputs, our method provides a more detailed understanding of the spatial distribution of NGPF, enabling more informed decision-making in farmland management practices.

To illustrate the practical utility of our method, we consider a scenario in which a government farmland management department is tasked with collecting annual farmland use information for a specific region and developing specialized farmland use plans. However, conducting large-scale manual field surveys and manually interpreting high-resolution remote sensing images is impractical and prone to subjective interpretation. In contrast, our method is straightforward to implement and requires only a minimal number of NGPF samples (obtained in the field or from low-precision grain crop data products) and easily accessible relevant open-source datasets, which are then input into the MaxEnt package. By implementing our method, government officials can accurately identify the fine distribution of NGPF and its influencing factors, enabling timely formulation of optimization strategies. In conclusion, our method improves the accuracy, efficiency, and applicability of NGPF identification, thereby contributing to more sustainable and efficient NGPF management. This, in turn, significantly contributes to achieving food security and promoting sustainable agriculture.

### 4.2. Comparison Between Our Results and Previous Findings

The NGPF identification results in this study are in good agreement with the results obtained from previous studies. For example, Sun et al. [37] revealed that the proportion of NGPF in Guangdong Province exceeds 45%. More importantly, the proportion of NGPF in Foshan was 86.18% in 2019 according to the Foshan Statistical Yearbook, which is highly consistent with the value of 88.90% reported in our study.

Regarding the influencing factors of NGPF, previous studies have suggested that elevation and slope, farming radius, population density and GDP can play important roles [12,18,37,63,85]. Our results are also consistent with these conclusions. First, our experiments show that the occurrence probability of NGPF first decreases and then stabilizes when the elevation increases. This is because flat farmland at low altitudes is generally close to urban areas, where farmers are more inclined to grow profitable crops for greater economic profit. Similar conclusions have also been drawn by Li et al. [30] and Zhao et al. [26].

Second, a greater probability of NGPF occurrence is observed in areas with a smaller farming radius. With an increase in the farming radius, the probability of NGPF first decreases, then increases, and finally stabilizes. This is because rural settlements are characterized by informal agglomeration and disorder, and the existence of discontinuous farmland among settlements (i.e., small farming radius) is unfavorable for growing grain crops. Farmers are more willing to grow vegetables and fruits on such farmland. As the farming radius increases (below a certain threshold), farmland becomes more concentrated and contiguous, and thus, the probability of NGPF decreases. Nevertheless, once the threshold is exceeded, the distance from farmland to rural settlements considerably increases, and farmers are more willing to grow non-grain crops that do not require frequent irrigation because of poor transportation infrastructure.

Third, when the CILUD increases, the occurrence probability of NGPF first increases and then decreases. On the one hand, the continuous increase in probability before reaching the peak is mainly due to the NGPF caused by the extensive degree of land use development. On the other hand, with the successful implementation of land use planning and farmland preservation policies, the probability of NGPF gradually decreases after the peak CILUD is reached.

Fourth, with an increase in population density, the occurrence probability of NGPF first increases rapidly, then decreases slowly, and finally stabilizes. When the population density is too low, the labor force for farming is insufficient such that crop production cannot be normally carried out. By comparison, the labor force increases as population density increases, and the probability of NGPF decreases correspondingly.

## 5. Conclusions

### 5.1. Findings and Policy Implication

Understanding the spatial pattern and associated influencing factors of NGPF can provide fundamental decision support for the prevention of NGPF. In this study, we innovatively combined multisource remote sensing images, natural/anthropogenic spatial influencing factors, and the MaxEnt model to fulfill these tasks at a grid scale (30 m resolution), in which the NGPF was regarded as a species. A case study in Foshan demonstrated that this method can accurately determine the spatial distribution of NGPF. According to our results, the NGPF in Foshan was mostly distributed in the northern and southwestern parts of Sanshui District and Nanhai District, the central and northern parts of Gaoming District, and the northern and central parts of Shunde District. Therefore, local authorities should pay enough attention to these areas.

More importantly, we found that elevation, farming radius, CILUD, and population density were the dominant factors affecting the spatial distribution of NGPF. Given the impact of elevation, farmland at lower altitudes should be carefully monitored to reduce the probability of NGPF occurrence. Local authorities should establish elevation-based agricultural zoning regulations that prioritize appropriate grain crop types for each altitude, with the aim of enhancing productivity and sustainability. Moreover, the impact of farming radius suggests that local authorities should also focus on farmland with extremely small or large farming radii, while providing guidance to farmers on the implementation of efficient agricultural practices tailored to the specific conditions of these radii. In fact, the distance from farmland to rural settlements should be reasonably redesigned. Furthermore, local authorities should maintain the CILUD of farmland at a low level and significantly improve land use efficiency during land development, both of which are conducive to reducing the occurrence of NGPF. Finally, regions with lower population density easily suffer from labor shortages, which eventually results in NGPF. Therefore, local authorities need to substantially increase agricultural subsidies for farmers to pro-mote the sustainable development of grain production. Our study provides valuable insights into agricultural sustainability and food security, contributing significantly to Sustainable Development Goal 2 (Zero Hunger). Additionally, the findings have significant implications for bolstering farmers’ social well-being and devising effective strategies for mitigating climate change, thereby contributing to the realization of SDG12 (Sustainable Consumption and Production) and SDG13 (Climate Action) to some extent.

### 5.2. Limitations and Future Research Directions

This study is subject to certain limitations that need to be addressed in future research. First, although multiple potential natural and anthropogenic influencing factors have been considered, the underlying causes of NGPF are far more complex in reality. Qualitative factors, such as local political dynamics, farming practices, government subsidies, agricultural policy decisions, and local customs, are not given sufficient attention in this study. These factors have the potential to exert a significant impact on the distribution of NGPF [33], and therefore future research should incorporate these factors to provide a more comprehensive understanding of their influence on NGPF patterns.

Second, while our method does not require the collection of negative samples, the accuracy of NGPF identification depends on the quality of the positive samples. The NGPF samples utilized in this study were extracted from the overlay of rice planting data and farmland distribution data. Although our results are satisfactory, the overlay of data from different production processes inevitably introduces a degree of uncertainty into this study. In future research, the use of grain crop data and farmland distribution data from the same source or the use of field surveys to obtain a limited number of NGPF samples could improve the quality of sample data while minimizing research interference.

Third, the grain crops included in this study were limited to rice, wheat, and corn. As highlighted by Zhang et al. [86], the perspective that potato should also be classified as a grain crop is increasingly gaining recognition among researchers [19]. If this perspective is adopted, the identification of NGPF in this study would exceed the actual occurrence. In subsequent research, it is also possible to include potato as a grain crop.

Fourth, this study focused on the fine-scale identification of NGPF and the investigation of influencing factors, and proposed concise governance strategies accordingly. However, the governance of the NGPF involves multiple aspects, such as policy formulation and implementation, which fall beyond the scope of the managerial insights provided in this study. The effective governance of excessive NGPF is an issue that deserves attention. Therefore, future research can focus on the management of NGPF to promote the sustainable development of grain production. The results of this study can provide crucial guidance toward this direction.

## Figures and Tables

**Table 1:** Proportion of NGPF in Foshan and its difference from the statistical yearbook.

District	Proportion of NGPF to Total Farmland Area of Foshan	Proportion of NGPF to Total Land Area of the District	Difference from Statistical Yearbook
Sanshui	28.00%	33.78%	8.61%
Nanhai	26.47%	24.67%	2.95%
Chancheng	0.93%	6.05%	0.73%
Gaoming	18.18%	19.36%	0.80%
Shunde	15.31%	18.96%	2.27%

**Table 2:** Contribution rate of each factor to the occurrence probability of NGPF (%).

Factor	Percent Contribution	Factor	Percent Contribution
Elevation	18.0	Band6	1.6
NDVI_Max4	16.9	Temperature in 2019	1.3
Farming radius	15.2	Band8	1.1
CILUD	12.8	Slope aspect	1.1
Band10	7.0	NDVI_Mean2	0.9
Population density	4.2	Precipitation in 2019	0.5
Band5	4.0	NDVI_Mean4	0.5
Residential land density	3.5	Traffic conditions	0.4
Irrigation conditions	2.0	Temperature (30 years)	0.4
Rainfall erosivity	2.0	Band1	0.4
GDP	2.0	NDVI_Max1	0.3
Slope	1.9	Potential evapotranspiration	0.3
Actual evapotranspiration	1.7		

Note (the same below): NDVI_Max denotes the average value of the monthly maximum value of the NDVI in a quarter, and the number denotes the quarter; NDVI_Mean denotes the average NDVI of the quarter; Band (number) denotes the number of bands in Landsat 8.

**Table 3:** Importance of each factor to the occurrence probability of NGPF (%).

Factor	Permutation Importance	Factor	Permutation Importance
Band10	13.4	NDVI_Max1	2.3
Elevation	10.5	Residential land density	2.1
Farming radius	10.4	Rainfall erosivity	2.0
Band5	8.0	Band8	2.0
GDP	6.2	Precipitation in 2019	1.7
Irrigation conditions	5.3	Potential evapotranspiration	1.5
Actual evapotranspiration	5.2	Slope aspect	1.4
Population density	5.0	Traffic conditions	1.4
CILUD	4.6	Band6	1.3
NDVI_Max4	4.0	Temperature in 2019	1.3
Slope	4.0	Band1	1.1
NDVI_Mean4	2.7	Temperature (30 years)	0.2
NDVI_Mean2	2.6		

## Data Availability

The original contributions presented in the study are included in the article/Supplementary Materials, further inquiries can be directed to the corresponding author.

## Supplementary Materials

The following supporting information can be downloaded at: https://www.mdpi.com/article/10.3390/foods13213385/s1, Figure S1: ROC curve and AUC of NGPF identification; Figure S2: ROC curve and AUC of NGPF results using only spectral information and NGPF results using only natural/anthropogenic information; Figure S3: Response curves between important factors and occurrence probability of NGPF; Table S1: Detailed information on the potential spatial influencing factors of NGPF; Table S2: Proportion of NGPF results using only natural/anthropogenic information in Foshan.
