# Supplementary material for: Investigating the Spatial Distribution and Influencing Factors of Non-Grain Production of Farmland in South China Based on MaxEnt Modeling and Multisource Earth Observation Data
Source: Foods. 2024 Oct 24;13(21):3385. doi: 10.3390/foods13213385 (PMC11545377; doi:10.3390/foods13213385)
Supplement: Supplementary file 1 [file foods-13-03385-s001.zip › foods-3241890-supplementary.pdf]

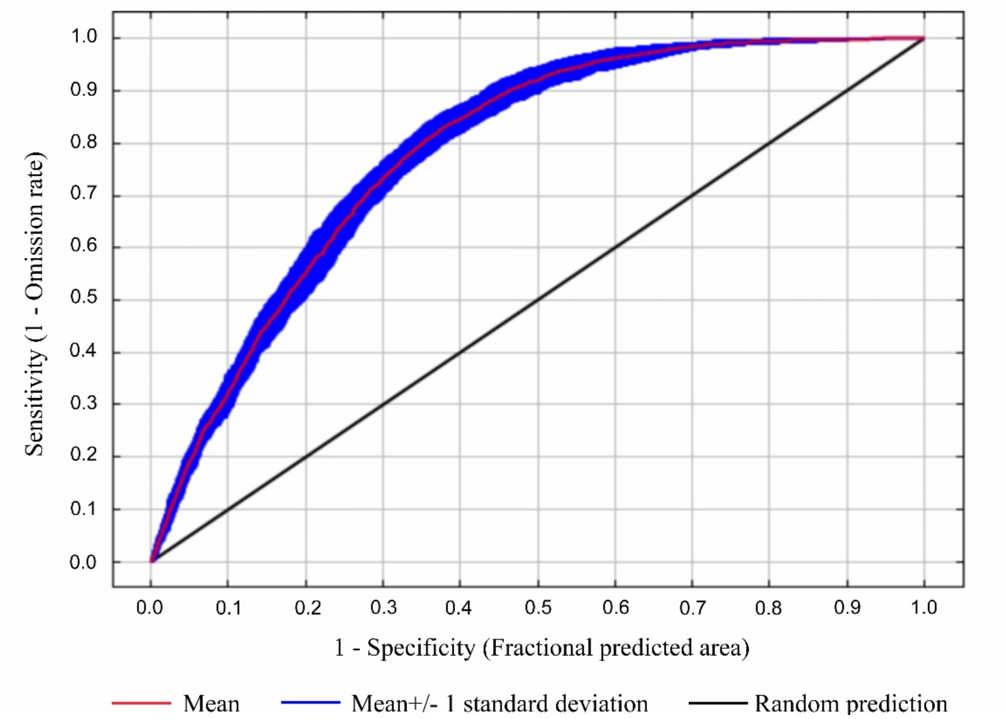

Figure S1. ROC curve and AUC of NGPF identification.

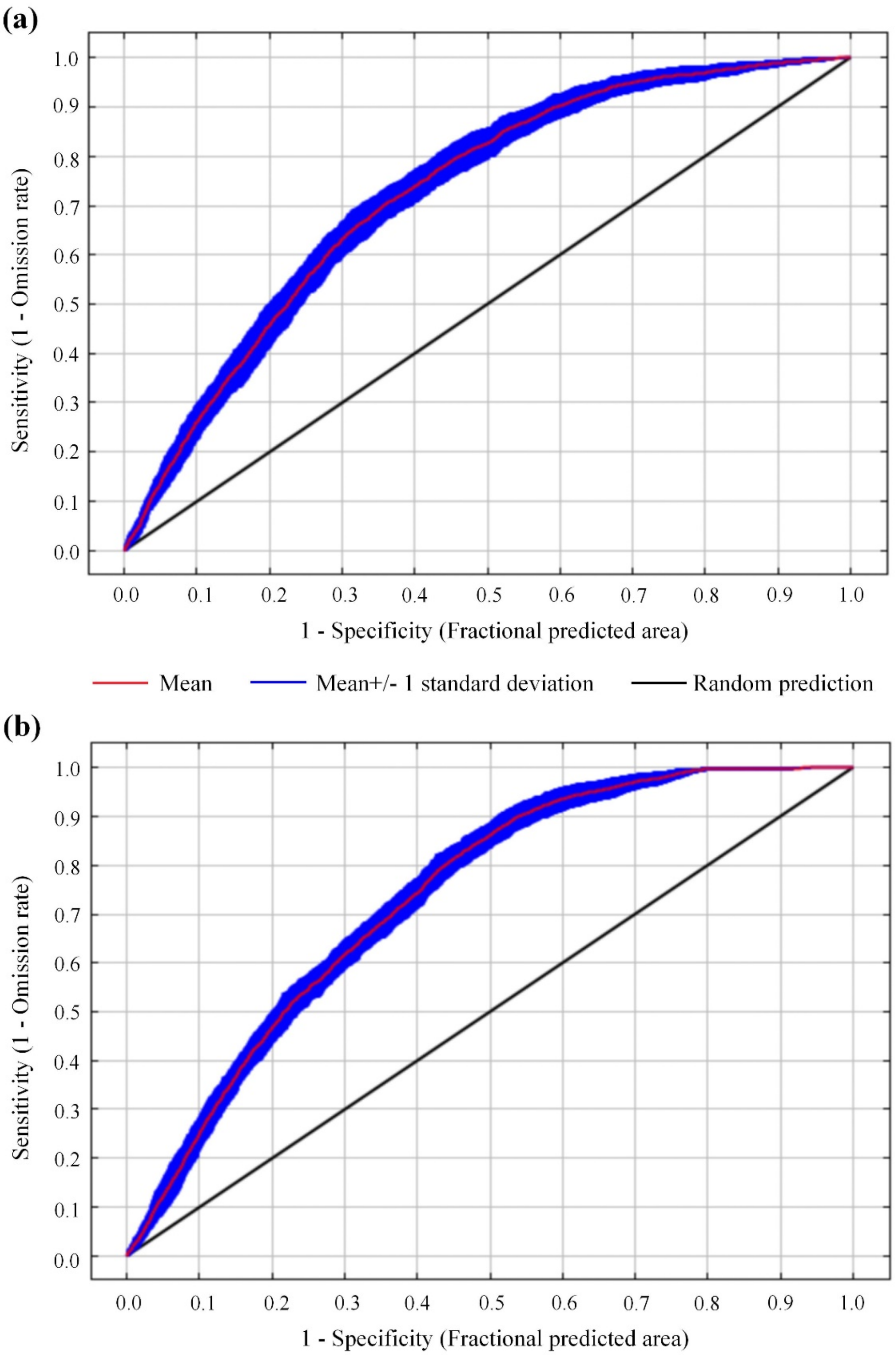

Figure S2. ROC curve and AUC of NGPF results using only spectral information and NGPF results using only natural/anthropogenic information.

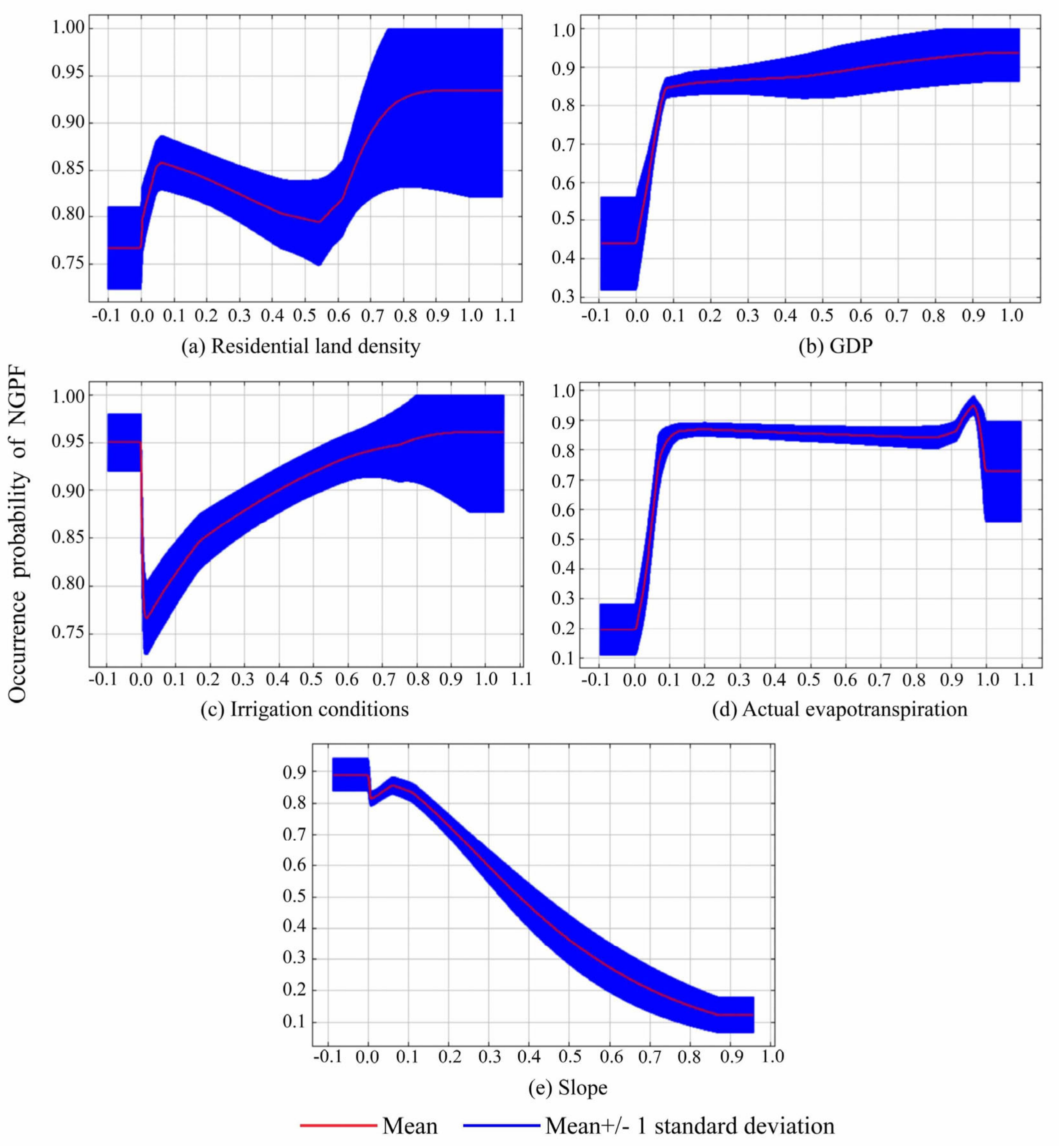

Figure S3. Response curves between important factors and occurrence probability of NGPF.

Table S1. Detailed information on the potential spatial influencing factors of NGPF.

Factor	Detail	Source
Elevation	Obtained from DEM	Geospatial Data Cloud Platform
Slope	Obtained from DEM	Geospatial Data Cloud Platform
Aspect	Obtained from DEM	Geospatial Data Cloud Platform
Precipitation in 2019	1 km spatial resolution, annual average	National Earth System Science Data Center
Temperature in 2019	1 km spatial resolution, annual average	National Earth System Science Data Center
Traffic conditions	Distance to major roads	OpenStreetMap
Irrigation conditions	Distance to water systems	OpenStreetMap
Farming radius	Distance to rural settlements	Science Data Bank
Population density	1 km spatial resolution	Science Data Bank
GDP	1 km spatial resolution	Science Data Bank
CILUD	1 km spatial resolution	Science Data Bank
Residential land density	1 km spatial resolution	Science Data Bank
Precipitation (30 years)	30-year average	Resource and Environment Science and Data Center, Chinese Academy of Sciences
Temperature (30 years)	30-year average	Resource and Environment Science and Data Center, Chinese Academy of Sciences
Potential evapotranspiration	30-year average	Resource and Environment Science and Data Center, Chinese Academy of Sciences
Actual evapotranspiration	30-year average	Resource and Environment Science and Data Center, Chinese Academy of Sciences
Rainfall erosivity	30-year average	Resource and Environment Science and Data Center, Chinese Academy of Sciences

Note: the spatial resolution of all raster data is 30 m except as noted; the year of data is 2019 except as noted.

Table S2. Proportion of NGPF results using only natural/anthropogenic information in Foshan.

District	Proportion of NGPF to total farmland area of Foshan	Difference from statistical yearbook
Sanshui	29.05%	9.65%
Nanhai	26.80%	2.62%
Chancheng	0.85%	0.65%
Gaoming	18.12%	0.86%
Shunde	13.60%	3.98%
